# Defining disordered eating trajectories across adolescence and assessing their impact on psychosocial outcomes using growth mixture modelling

**DOI:** 10.1186/2050-2974-1-S1-O65

**Published:** 2013-11-14

**Authors:** Sarah A Mitchell, Leah Brennan, Isabel Krug, Nicholas B Allen

**Affiliations:** 1 School of Psychology and Psychiatry, Faculty of Medicine, Nursing and Health Sciences, Monash University, Australia; 2 School of Psychology, Australian Catholic University, Australia; 3 School of Psychological Sciences, University of Melbourne, Australia; 4 Orygen Youth Health Research Centre, Centre for Youth Mental Health, University of Melbourne, Australia

## 

Few studies have examined different trajectories of disordered eating in adolescence across time which has particular relevance given the extensive maturation during this period.

## Aims

To identify trajectory classes of disordered eating across adolescence using Growth Mixture Modelling and to examine differences in psychosocial outcomes (lifetime history of mood, anxiety and substance use disorders, sleep quality, depression and anxiety symptoms, rumination and general psychosocial functioning) between classes.

## Method

Participants included 213 individuals (106 males) from 3 waves of the Adolescent Development Study; Time 1 (mean age=14.98), Time 2 (mean age=16.61) and Time 3 (mean age=18.85).

## Results

Factor scores based on confirmatory factor analysis of subscales and behavioural frequency items from the EDE-Q were used to estimate trajectories. The best fitting model comprised a 4-Class model including "high decreasing, moderate increasing, low stable, and low decreasing" trajectories. Further, the high decreasing and moderate increasing trajectories were characterized by poorer psychosocial outcomes compared to the remaining two classes (p-values ranged from <.001 to <.05 for the majority of comparisons).

## Conclusions

Findings provide increased insights into different developmental patterns of disordered eating across adolescence and suggests that even if symptoms decrease across time, they are still associated with poorer outcomes during late adolescence.

This abstract was presented in the **Understanding and Treating Eating Pathology** stream of the 2013 ANZAED Conference.

